# On the Prolonged Use of Bromide of Potassium, in Large Doses, and Its Value in the Treatment of Epilepsy

**Published:** 1869-05-01

**Authors:** J. N. Danforth

**Affiliations:** Chicago


### CHICAGO MEDICAL JOURNAL.

Vol. XXVI[illegible]—MAY 1, 1869.—No. 9.

### ORIGINAL COMMUNICATIONS.

### *On the Prolonged Use of Bromide of Potassium, in Large Doses, and its Value in the Treatment of Epilepsy.*

BY J. N. DANFORTH, M.D., CHICAGO.

The past ten years have proved unusually prolific as regards new remedies; and of these no one has obtained a better reputation, or more rapidly grown in favor with the medical profession than the bromide of potassium.

The experiments of Locock, Brown-Sequard and Behrend, long ago established the value of this remedy in cases where obstinate wakefulness or "coma vigil" was the predominating morbid condition; and this without very much regard to the essential or underlying cause thereof. But a more extended and comprehensive experience has likewise proved that the bromide of potassium is an exceedingly valuable remedy in many of the chronic diseases which directly involve the great nervous centers. Prominent among these may be mentioned epilepsy—both on account of its obscure pathology, and the sadly inefficient means for its treatment, heretofore at our command. Indeed, from the earliest dawn of medicine as an inductive science, up to the present period of active research and discovery, the unfortunate epileptic has been universally abandoned as hopelessly incurable.

Dr. Watson (Practice of Physic, page 425), after enumerating the formidable array of remedies which have been successively recommended, tried and condemned, says: "This long array of drugs, all of which have been known, or supposed, to accomplish a cure, affords, in truth, one of the strongest evidences of the intractability of the disease under any plan of treatment"—a remark, the truth of which, every physician of any considerable experience has too often felt himself obliged to assent to.

Latterly, however, the bromide of potassium has seemed to outrank all other remedies in the treatment of this fearful disease—having been brought prominently before the profession by the writings of Dr. J. Russell Reynolds, of University College, London, and of Drs. Bazire, Brown-Sequard, Rannkill, Radcliffe and Hughlings Jackson, of the British National Hospital for the Paralyzed and Epileptic. The experience of these eminent physicians seems to indicate pretty clearly that, in order to derive any real, or at least permanent benefit from this drug two conditions are always and absolutely essential: 1st. that the remedy be given in large doses; 2d. that its administration be persistently and uninterruptedly continued, for months, or even *years*, if a cure be not sooner accomplished. As this course contemplates the administration of a remedy of acknowledged and well-established potency in doses larger, and for a period of time longer than has been, or is now deemed altogether safe by the great majority of physicians, I propose to raise, and attempt, as far as I am able, to answer, these two queries: First, to what extent may the bromide of potassium (or ammonium, for one of these salts may be looked upon, as, in its therapeutic relations, the type of the other) be given without endangering the safety of the patient? and, secondly, How long may these unusually large doses be continued, without producing injurious or alarming results?

First, as to the dose. An ordinary dose of the bromide is stated to be "from three to ten grains, three times a day;" but this quantity, as experience has abundantly and repeatedly proved, is utterly inefficient and useless in the treatment of epilepsy in the adult. On the other hand, the experience of many eminent and trustworthy observers, in different quarters of the globe, seems to have demonstrated, beyond dispute, that this remedy, given in much larger doses, as from fifteen to sixty grains twice or thrice daily, *does* exercise a marked control over the epileptic attacks, whatever may be said in regard to a permanent and unqualified cure.

It follows, then, that these somewhat startling doses *must* be given, or the patient *must* be abandoned to his fate, and epilepsy must continue to be, as it has been, the opprobium of medicine. The terrible nature of this disease, and the almost inevitable mental ruin in which it terminates, demand of us the employment of every efficient measure at our command; nor should the ill success of the past, or prejudice, or merely theoretical notions as to its incurability, be allowed to stand in the way of the careful, faithful and prolonged trial of any and every remedy which seems to promise the least benefit to this most unfortunate class of patients.

In the present instance, the *real* truth seems to be, that the bromides have been repeatedly given in doses of from fifteen to forty, or even sixty, grains, not only without any perceptibly injurious effects, but with the happiest results, especially in the treatment of epilepsy; that this practice is daily gaining ground with those physicians whose opportunities for acquiring experience in the treatment of epilepsy are such as to be of the greatest value, and command the fullest confidence; and that the practitioner has hardly the right to withhold this remedy, in cases where the epileptic attacks are sufficiently frequent to demand treatment at all.

Probably the National Hospital for the Paralyzed and Epileptic has, heretofore, afforded the widest and most fruitful field for observation in regard to the true merits of the bromides; and the testimony of the officers of this institution are very pointedly in their favor. Says Dr. P. Victor Bazire (Trousseau, Vol. I, p 99, note), "At the National Hospital for Paralysis and Epilepsy, bromide of potassium has been extensively used by Dr. Brown-Sequard, Dr. Ramskill, Dr. Radcliffe, Dr. Hughlings Jackson and myself." * * * These gentlemen speak of the remedy in terms of unqualified praise. They are in the habit of using it in doses of from fifteen to twenty or thirty grains twice or thrice daily—or in accordance with the demands of each individual case. The dose most commonly employed by them, however, is fifteen or twenty grains twice a day, given when the stomach is empty; and in this quantity, writes Dr. Bazire, "I have seen patients who had taken the bromide for two and three years" without any injurious effects whatever. But Dr. J. Russell Reynolds is, perhaps, the most earnest advocate of large doses. "Bromide of Potassium," writes he, "in small doses, has appeared to be of little or no service; but in large doses it has scarcely ever failed to give much relief;" (System of Medicine, Vol. 2, p 280,) and, again (op. cit. p 968), "the cure of epilepsy is effected by doses varying from five to forty grains, given three times daily;" and the latter dose, he adds, "does no harm, for it may be taken for many months and even for years, without producing derangement of any sort, or in any direction." M. Behrend relates a case in the *American Medical Times*, Vol. 6, p 45, in which he gave twenty-five grains three times a day for more than a week, with the happiest effects, and adds that "it produces in these large doses neither disagreeable nor toxical effects." Dr. Samuel R. Percy, of New York, has given "eight drachms in seventy-two hours, without headache or other unpleasant symptoms."

It is not my design, however, to enter the lists as the champion of any system of dosing, large or small, or to present a one-sided and partisan view of a subject of such practical and growing importance; but rather to collate and briefly state the facts on both sides. While, therefore, it seems to be well established that the bromide may be safely and beneficially given in very large doses in the great majority of cases, it is also true that unpleasant and injurious effects have, occasionally, been met with. The symptoms indicative of an over-dose, or of "saturation" of the system, are those which are usually recognized as specific of bromine, or "bromism." There is debility, sluggishness, stupor, drowsiness, hebitude. The conjunction becomes insensible, the pupil is dilated, the sense of taste is impaired, and general torpidity of the various functions ensues. Directly "disorders of motility manifest themselves;" the patient "lounges about, his gait becomes altered, and he staggers and rolls like a drunken man." (Bazire.) These symptoms disappear, however, if the medicine be suspended, and some mild stimulant is substituted. Dr. J. Russell Reynolds states, in his usual precise manner, "having seen given K Br. to many hundred of individuals, I have witnessed no ill effects from its administration. I say advisedly *no* ill effects, for in only three cases have there occurred any unpleasant symptoms which could by any possibility be referred to the drug." In one of these three "was some swelling of the nose, and of the submaxilary glands;" in another an "irritable vesicular eruption appeared upon the skin;" these symptoms rapidly disappearing, in both cases, directly the drug was suspended. In a third case there was "the recurrence of syncopal sensations, which subsided on the addition of twenty drops of chloric ether to each dose of the bromide." (System of Med. Vol. 2, p 281.) But these effects are comparatively rare; are slowly developed, and are exceedingly trivial when com pared with the benefit which almost certainly follows the administration of the remedy; and, as already stated, they invariably disappear if the medicine be withheld for a few days.

Secondly: How long may the administration of K Br. in large doses be continued with safety? A partial answer to this question has already been given; a more complete response, however, may be found in the positive statements of the writers already quoted. Dr. Reynolds insists that the remedy may, with entire safety, be given in large doses for any length of time—"for many months, and even for years;" and that "the dose should be gradually increased; and the exhibition of the drug should be most patiently carried on, until all indications for its use shall have ceased. The rash, or acne, on the skin which, is occasionally seen, "is not determined by the quantity of the bromide that is taken;" it sometimes appears after a few doses of five grains each have been taken, "and it has been absent in many cases where thirty grains have been taken three times daily, for periods of six or even twelve months." Bazire, with equal confidence, says: It is remarkable how long moderate doses of the drug can be administered without sensibly affecting the system." "I have seen patients," continues he, "who had taken the bromide for two and three years, in doses of from ten to fifteen grains, two, and sometimes three times a day, with occasional short intermissions only, and whose general health had not, apparently, suffered." It would be perfectly easy to extend these extracts—all strongly corroborative of those already given—to almost any extent, but it scarcely seems necessary.

The conclusion is just and fair that the bromide may, with safety, be administered in large doses, as high, even, as thirty grains thrice daily, if need be, in the treatment of epilepsy; that it should be persistently given for many weeks and months; that its effects should be carefully watched; that it should be suspended the moment any symptoms of "saturation" or of "bromism" appear; but that it should be immediately resumed in smaller doses, as soon as these symptoms disappear. To this it may be added that the amount demanded in each individual case can, generally, and probably always, be determined, after a few weeks of careful observation. Indeed, it is quite possible that the best effects of bromide in the treatment of epilepsy are only reached when the system is brought under its influence to a point just short of actual "bromism."

What is the real value of bromide of potassium, in the treatment of epilepsy? Does it accomplish a thoroughgoing and permanent cure, or is it merely palliative?

It is very difficult to frame an answer to these queries which shall cover the whole truth. In the present state of our knowledge, and with the comparatively limited experience which physicians have, up to the present time, had in the use of this remedy, it is quite impossible to say precisely how much may be expected from it. But the following propositions are probably not far from the truth:

1st. In a limited number of cases, no good effects can be perceived, even after the remedy has been persistently taken for weeks, or even months.

2d. In a very small proportion of cases, positive cures have resulted; that is, confirmed epileptics have been absolutely free of attacks for a period of time which warrants the conclusion that an absolute cure has been accomplished; and this even, after having discarded the remedy for many months.

3d. In a very large proportion of the cases submitted to systematic and careful treatment with the bromides, great benefit has resulted; in many the attacks are entirely prevented, so long as the medicine is regularly taken; in others the attacks have become very much less frequent, and less persistent, while great improvement in the bodily health, and a more cheerful and hopeful state of mind have likewise resulted; in still others the improvement has been much less, but sufficient to encourage both patient and physician to persevere in the use of the remedy. In fact, an absolute cure, on the one hand, and absolutely no benefit, on the other hand, seem to be the rare exceptions; while in the great mass of cases, the patient receives very marked benefit, in that the attacks are rendered very much less frequent, or altogether suspended during the employment of the medicine; in that the general health usually undergoes a marked improvement; and in that cheerfulness and vivacity take the place of despondency, gentleness and patience supplant irritability and moroseness, and the whole demeanor of the patient becomes changed and improved. These are the results which have been obtained by those who have had the greatest experience in the use of the remedy in question. Bazire says: "It is infinitely superior to all other remedies that have been recommended against epilepsy." Dr. C. Bland Radcliffe is equally emphatic in his testimony, namely: * * * * "The conclusion at which I have arrived is, that bromide of potassium is the only remedy, in epilepsy, upon which much reliance can be placed." Dr. J. Russell Reynolds, however (who is, *par excellence*, the advocate of large doses), seems to have met with the best results. After alluding to its power as a palliative, he adds, "in some cases, it has completely cured the patient, and the cure has been permanent for years, *and is so now.* Brown-Sequard, Duckworth Williams, Ramskill and Hughlings Jackson are no less earnest in their advocacy of the claims of this remedy. It is useless, however, to summon a cloud of witnesses to sustain a point already beyond dispute.

My own experience in the use of K Br. as a remedy for epilepsy has been somewhat limited. A long life-time of private practice, even in a populous city, can be expected to furnish comparatively few cases of this most distressing malady; and it is very difficult to retain cases under treatment sufficiently long to accomplish much good by any remedy—much less a remedy which the patient is told, at the outset, he must take for "many months, perhaps for years." I may be permitted, however, to conclude this paper by the relation of a single case, which must stand as the type of several others which have come under my observation. In doing this, I shall state the facts as briefly as possible, merely premising that it was a case of true and unmitigated epilepsy, of long standing.

Case.—Peter M., aged twenty, consulted me in Sept., 1867, for epilepsy. Ten years previously, while living in Canada, he drank the contents of the camphor bottle, and ate the undissolved camphor remaining in the bottle. For twenty-four hours afterwards, according to his mother's account, he was comatose, and exceedingly pale, "almost like death." After this he vomited freely, when the symptoms improved, and he slowly recovered. In less than a month he had a severe attack of epilepsy (this being his first attack), and from this time onward the attacks recurred with increasing frequency, until, when I first saw him, the intervals were never longer than a month. The eyes were dull and listless, the countenance vacant, the memory defective, and the aspect of the patient was that of semi-imbecility. I prescribed ten grains of bromide of potassium three times a day. In the course of the next two months he had two attacks. The dose was then increased to fifteen grains, three times a day. He shortly after had another "fit," after which they disappeared, and for fifteen months succeding he suffered not a single attack. Meantime his general appearance improved; the countenance assumed a more intellectual aspect, he became more vivacious; and he even sought, and obtained, employment, giving satisfaction in the capacity of fireman to a stationary engine. At the end of the time last mentioned, he began to act as "night-watchman," and ceased taking the medicine with regularity. The result was that before a month had passed, he had a recurrence of epilepsy. The bromide was now resumed, and the epileptic attack again ceased. After taking the remedy for several weeks, without any interruption, swelling of the submaxilary glands and soreness of the throat came on; the medicine was suspended for a few days, when these symptoms at once disappeared. But another epileptic seizure was the penalty he paid for the temporary suspension of the remedy. Bromide of ammonium was now substituted for K Br., in doses of fifteen grains three times a day, and he has constantly taken one of these salts in quantities varying from forty-five up to sixty grains a day, up to the present time. No injurious or unpleasant effects, beyond those already mentioned, have, at any time, appeared; on the other hand, the general health has steadily improved, and the patient has, for many months, pursued his employment without interruption.

This case affords an excellent illustration not only of the *good*, but of the ill effects of the bromides in the treatment epilepsy; and of the results which we may reasonably expect in the great majority of instances. But this result, even, is well worth achieving. Any remedy which will, to any extent, mitigate the horrors of this dreadful disease, is, indeed, a boon and a blessing to its unfortunate victims.

